# Prevalence and factors associated with smoking among adults in Malaysia: Findings from the National Health and Morbidity Survey (NHMS) 2015

**DOI:** 10.18332/tid/82190

**Published:** 2018-01-26

**Authors:** Kuang H. Lim, Chien H. Teh, Sayan Pan, Miaw Yn Ling, Muhammad F. M. Yusoff, Sumarni M. Ghazali, Chee C. Kee, Kuang K. Lim, Kar H. Chong, Hui L. Lim

**Affiliations:** 1 Institute for Medical Research, Kuala Lumpur, Malaysia; 2 Institute of Public Health, Kuala Lumpur, Malaysia; 3 Hospital Sultan Haji Ahmad Shah, Pahang Darul Makmur, Malaysia

**Keywords:** smoking, Malaysian adults, social-demographic factors, NHMS

## Abstract

**INTRODUCTION:**

The continuous monitoring of smoking prevalence and its associated factors is an integral part of anti-smoking programmes and valuable for the evaluation of the effectiveness of anti-smoking measures and policies. This study aimed at determining prevalence of smoking and identifying socio-demographic factors associated with smoking among adults in Malaysia aged 15 years and over.

**METHODS:**

This is a cross-sectional study with a representative sample of 21 445 adults in Malaysia, aged 15 years and over, selected via a stratified, two-stage proportionate-to-size sampling method. Data were obtained from face-to-face interviews by trained research assistants, using a standard validated questionnaire. Multivariable logistic regression was performed to determine socio-demographic factors associated with smoking among Malaysians.

**RESULTS:**

The overall prevalence of smoking was 22.8% (95% CI: 21.9–23.8%), with males having a significantly higher prevalence compared to females (43.0%, 95% CI: 41.1–44.6 vs 1.4%, 95% CI: 1.1–1.7). The highest smoking prevalence was observed among other ethnicities (35.7%), those aged 25–44 years (59.3%), and low educational attainment (25.2%). Males, those with lower educational attainment and Malays were significantly associated with smoking.

**CONCLUSIONS:**

The prevalence of smoking among Malaysians, aged 15 years and over, remains high despite the implementation of several anti-smoking measures over the past decades. Specially tailored anti-smoking policies or measures, particularly targeting males, the Malays, younger adults and those with lower educational attainment, are greatly warranted to reduce the prevalence of smoking in Malaysia.

## INTRODUCTION

Morbidity and mortality related to smoking is a major public health challenge worldwide. Approximately 3 million deaths are reported each year and 10 million mortalities from smoking-related diseases are expected globally by 2030. About 70% of these mortalities are expected from developing countries due to high prevalence of smoking^1-3^. Malaysia is not spared from this smoking scourge, where 10 000 deaths^4^ attributed to smoking are reported each year and smoking-related diseases have been identified as the major contributor to disability-adjusted life years and lost years of life among the Malaysian population^5,6^. If the current trend in smoking persists then 30 000 deaths are expected annually by the year 2020^4^.

The Ministry of Health in Malaysia has initiated and implemented several anti-smoking policies and measures to reduce the prevalence of smoking among the Malaysian population via legislation, health promotional activities, and community intervention programmes^7,8^. These measures were in line with the MPOWER measures introduced by the World Health Organization Framework Convention on Tobacco Control^9^ and were rectified by Malaysia in 2005 with the ultimate aim to reduce public health problems related to smoking. In addition to active anti-smoking measures, regular monitoring of smoking prevalence to evaluate the effectiveness of anti-smoking measures is also a critical component in the endeavour to combat smoking.

A series of nationwide studies such as the National Health and Morbidity Survey (NHMS) conducted in 1986, 1996 and 2006, as well as the Global Adult Tobacco Survey (GATS) conducted in 2011^10-13^, were mechanisms to monitor the smoking prevalence among Malaysians. The latest GATS conducted in 2011 across all States in Malaysia revealed that almost a quarter of Malaysian adults were current smokers. Prevalence of smoking was significantly higher among males, younger respondents (25-44 years) and those with lower educational attainment^13^. Nonetheless, in view of the rigorous anti-smoking efforts of the Malaysian government, especially during recent years, there might be some changes in the current smoking situation in Malaysia. Therefore, the present paper extracted data from the latest NHMS in 2015 to determine the prevalence of smoking and its associated factors among a representative sample of adults in Malaysia, aged 15 years and over, since up-to-date evidence-based findings are important input for proper and specially tailored anti-smoking policies and programmes.

## METHODS

### Study sample

The National Health and Morbidity Survey 2015 was a cross-sectional study of morbidity, health status and health-seeking behaviour among a nationally representative, non-institutionalised general population of adults in Malaysia, aged 15 years and over. These representative respondents were selected via a stratified, two-stage sampling method. The first-stage stratification involved 15 States in Malaysia, and the second-stage stratification comprised urban and rural areas for each State. The urban area is defined as a gazetted administrative area (a carefully mapped area with definite boundaries that have been published in the Government Gazette for public information) with adjoining built-up areas of 10 000 people or more, while a gazetted administrative area of less than 10 000 people is defined as a rural area^14^. The first stage sampling involved a random selection of 869 Enumeration Blocks (EBs) as primary sampling units (643 urban and 333 in rural EBs) from all EBs in Malaysia via a probability-proportional-to-size sampling technique. An EB is an artificial geographical area consisting of 80-120 Living Quarters (LQs), created by the Malaysian Department of Statistics (DOS) according to the 2010 census. Subsequently, 12 Living Quarters (LQs) were selected in each selected EB during the second-stage sampling. Finally, all households and eligible household members in the selected LQs were recruited for the study. The study protocol was approved by the Medical Research and Ethics Committee (MREC), Ministry of Health Malaysia.

### Data collection

Selected respondents were interviewed face-to-face by trained research assistants, using a standardised questionnaire. Informed consent was obtained from all respondents before the interview. To ensure a high response rate, respondents who were not at home, or LQs that were found vacant, during the initial visit were re-visited up to three times. If the respondent was still unreachable after the 3 attempts, they were excluded from the study.

### Questionnaire

The NHMS 2015 questionnaire involved 17 topics, including smoking. The smoking questionnaire adopted a subset of key questions from the GATS 2011^14^ that had 8 components: namely socio-demographic background, smoking status, type of tobacco product used, exposure to secondhand smoke (SHS) at home and the workplace, intention to quit, exposure to tobacco product advertisement, and information on anti-smoking campaign.

The smoking status of respondents were determined by the answers (‘daily’, ‘less than daily’, and ‘not at all’) to two items: ‘Do you currently smoke tobacco?’ and ‘Do you currently use any smokeless tobacco?’. Respondents who answered ‘not at all’ to both items were categorised as ‘non-smokers’ whilst those who answered ‘daily’ or ‘less than daily’ to either one or both items were considered as ‘current smokers’.

Socio-demographic background such as gender (male, female), ethnicity (Malay, Chinese, Indian, Οther Bumiputras, Others), educational attainment (no formal education, primary, secondary, tertiary education), age group (15-24, 25-44, 45-64, 65 years and over), locality (urban, rural), income level (quintile one to five), marital status (single, married, widow/widower/separated) and occupation (government employee, private employee, self-employed, homemaker and retiree) were also included in the questionnaire. Other Bumiputras included Iban, Kadazan, Dusun, Bidayuh, Melanau and Bumiputras from the State of Sabah and Sarawak, as well as the Orang Asli, whereas ‘Others’ included Serani, Sikh, Siamese, Indonesia, Suluk, and other foreigners.

### Data analysis

The sample was weighted to represent the general population aged 15 years and over, based on the 2010 Malaysia Population Census. Descriptive statistics were used to illustrate the socio-demographic characteristics of the respondents, as well as the proportion of smokers by socio-demographic characteristics. Multivariable logistic regression was used to determine the association between each socio-demographic factor and smoking status, after adjustment for other confounding factors. Finally, the fit of the final model was confirmed by STATA Version 11 statistical software via a modified Hosmer-Lemeshow Goodness-of-Fit test for complex sampling^15^ with a non-significant p value (>0.05). Tests for possible two-way interactions in the final custom model showed no significant interactions were present. Data were presented with 95% confidence intervals without p values, as the large sample size in the present study could generate significant results even if statistical differences or associations are small.

## RESULTS

A total of 51.5% of respondents were males. Most of the respondents were urban dwellers (75.8%), aged 25-64 years (77.3%), married (60.3%) and of Malay descent (49.6%). More than half of the respondents had either attended a primary or secondary school (68.7%), whilst only one-fourth attained a tertiary educational level (24.8%). Almost half of the respondents worked in the private sector (47.8%) (Table 1).

**Table 1:** Social demographic characteristic of respondents

*Variable*	*Estimated population*	*n*	%	*95% Confidence Interval*	
				**Lower**	**Upper**
**Sex**				
Male	11305164	10220	51.6	50.7	52.4
Female	11610444	11225	48.4	47.6	49.3
**Ethnicity**				
Malay	10872208	13345	49.6	46.7	52.5
Chinese	5087215	3407	23.2	20.8	25.8
Indian	1480516	1519	6.8	5.8	7.9
Other Bumiputras	2394459	1891	10.9	9.2	13.0
Others	2081208	1283	9.6	7.9	11.4
**Age group (years)**				
15-24	5493294	4219	25.1	24.1	26.1
25-44	9355186	7984	42.7	41.4	44.0
45-64	5395163	6793	34.6	23.8	25.5
65 +	1671964	2449	7.6	7.1	8.2
**Income level**				
Quintile 1	2570888	2978	11.7	10.8	12.7
Quintile 2	3731827	4008	17.0	16.0	18.1
Quintile 3	4545777	4661	20.7	17.5	22.0
Quintile 4	4792572	4431	21.9	20.5	23.3
Quintile 5	6274541	5367	28.6	26.8	30.5
**Education attainment**				
No formal education	1202008	1385	5.5	4.9	6.2
Primary	4368078	5015	20.0	18.9	21.1
Secondary	10642592	10294	48.7	47.4	49.9
Tertiary	5284860	4403	24.2	22.8	25.6
**Marital Status**				
Married	7278630	5645	33.2	32.2	34.4
Single	13209903	13845	60.3	59.2	61.5
Divorce/widow/er	1404922	1941	6.4	6.0	6.9
**Occupation**				
Government	1925402	2195	11.3	10.3	12.4
Private	8135063	6204	47.8	46.1	49.1
Self employed	3448862	3885	26.3	19.1	21.4
Unpaid/homemaker	2909014	3347	17.1	16.3	17.9
Retiree	609305	786	3.6	3.2	4.0
**Residential**				
Urban	16609048	12369	75.8	72.0	78.6
Rural	5306560	9076	24.2	21.4	27.2

Approximately 5 million Malaysian adults (22.8%), aged 15 years and over, were current smokers. The prevalence of current smokers was significantly higher in males (43.0, 95%CI: 42.0-44.6) compared to females (1.4%, 95%CI: 1.0-1.8), as a whole and across all socio-demographic groups. The Chinese (14.2%, 95%CI: 12.7-15.9) and Indians (16.5%, 95%CI: 13.9-19.4) had a significantly lower prevalence of smoking compared to other ethnic groups. Adults aged 25- 44 years (28.3%, 95%CI: 26.9-29.8) reported the highest prevalence of smoking, but those with tertiary educational attainment (14.9%, 95%CI: 13.5-16.3) and those with an income level at the lowest (16.5%, 95%CI: 14.6-18.6) or highest (19.3%, 95%CI: 17.7- 21.1) quintile had significantly lower prevalence of smokers. On the other hand, the smoking prevalence was significantly higher among the self-employed workers (35.4%, 95%CI: 33.2-37.6) and those who worked in the private sector (31.7%, 95%CI: 29.8-33.6), compared to government servants, retirees and homemakers (Table 2).

**Table 2:** Smoking status by social-demographics among Malaysian population aged 15 years and over

*Variable*	*Current smoking status 2015*			
	*Estimated population*	*n*	%	*95% CI*
Overall			22.8	
**Sex**				
Male	4847892	4351	43.0	41.1-44.6
Female	143566	126	1.4	1.1-1.7
**Ethnicity**				
Malay	2686373	2970	24.7	23.6-25.9
Chinese	719321	460	14.2	12.7-15.9
Indian	244131	220	16.5	13.9-19.4
Other Bumiputras	613563	451	25.8	23.2-28.6
Others	728167	376	35.0	31.1-39.2
**Age group (years)**				
15-24	1068902	793	19.5	17.7-21.4
25-44	2646668	2025	28.3	26.9-29.8
45-64	1085008	1325	20.1	19.0-21.4
65 +	190878	334	11.5	9.8-13.3
**Income level**				
Quintile 1	420632	456	16.5	14.6-18.6
Quintile 2	999915	941	26.8	24.8-28.9
Quintile 3	1130301	1091	25.1	23.3-27.0
Quintile 4	1221197	1017	25.5	23.6-27.5
Quintile 5	1211680	972	19.3	17.7-21.1
**Education attainment**				
No formal education	258514	216	21.5	17.6-26.1
Primary	1099147	1086	25.2	23.3-27.2
Secondary	2740921	2475	25.8	24.5-37.1
Tertiary	785981	637	14.9	13.5-16.3
**Marital Status**				
Married	1713143	1276	23.6	21.9-25.4
Single	3141651	3022	23.8	22.7-24.9
Divorce/widow/er	136663	179	9.7	7.9-11.9
**Occupation**				
Government	445344	431	23.1	20.6-25.9
Private	2574645	1898	31.7	29.8-33.6
Self employed	1220582	1348	35.4	33.2-37.6
Unpaid/homemaker	61011	68	2.1	1.5-2.9
Retiree	117466	168	19.3	16.0-33.1
**Residential**				
Urban	3515923	2373	21.2	20.0-22.4
Rural	1475534	2104	27.9	26.2-29.6

Cigarette was the main tobacco product used/smoked by current smokers (87.9%, 95%CI: 86.4-89.3), followed by smokeless tobacco (47.1%, 95%CI: 44.2–50.0). Only a small portion of smokers used pipe and cigar. The preferred types of tobacco product did not differ significantly by gender. However, on average, male smokers smoked a significantly higher number of cigarettes (20.7, 95%CI: 19.4-21.9) per day compared to female smokers (13.7, 95%CI: 9.8-17.6) (Table 3).

**Table 3:** Type of tobacco product used and number of cigarettes smoked daily by Malaysian adults aged 15 years and over, by gender

*Type of tobacco used*	*Estimated population*	*n*	%	*95% CI*
Any smoked tobacco product	4935109	4418	22.6	21.6-23.5
Smokeless tobacco	2393112	2166	10.9	10.1-11.7
Hand-rolled	503862	684	2.3	2.0-2.6
**Cigarette smoked daily (sticks)**				
1-5	987759	980	24.4	22.4-26.6
6-10	1016431	869	25.1	23.2-27.1
11-15	322083	268	8.0	6.9-9.2
15-20	773702	738	19.1	17.5-20.8
21 and above	947704	806	23.4	21.1-25.9

The multivariate logistic regression revealed that the likelihood of smoking was significantly higher among males than females. The Malays (aOR 2.43, 95%CI: 2.02-2.93) and other indigenous groups (aOR 2.00, 95%CI: 1.53-2.62) were more probable of becoming a smoker compared to other ethnic groups. The likelihood of being a smoker was highest among those aged 25-44 years (aOR 3.92, 95%CI: 3.06-5.47) and those with lower educational attainment, and a comparable likelihood of smoking was also found among those who earned the least or the most (Quintile 5). Self-employed (aOR 1.86, 95%CI: 1.42-2.45) and private workers (aOR 1.80, 95%CI: 1.30-2.37) were also found to have higher odds to be smokers. However, no significant associations between marital status, locality and smoking were observed (Table 4).

**Table 4:** Association between social-demographic factors and smoking among Malaysian adults aged 15 years and over

*Variable*	*Adjusted Odds Ratio*	*95% CI*	
		Lower	Upper
**Sex**			
Male	57.92	41.00	80.41
Female	Ref		
**Ethnicity**			
Malay	2.43	2.02	2.93
Chinese	Ref		
Indian	1.11	0.81	1.54
Other Bumiputras	2.00	1.53	2.62
Others	1.86	1.40	2.48
**Age group (years)**			
15-24	3.10	2.11	4.56
25-44	3.92	2.95	5.22
45-64	2.05	1.56	2.70
65 +	Ref		
**Income level**			
Quintile 1	1.28	0.93	1.64
Quintile 2	1.30	1.07	1.58
Quintile 3	1.22	1.01	1.47
Quintile 4	1.33	1.11	1.59
Quintile 5	Ref		
**Education attainment**			
No formal education	3.28	2.27	4.75
Primary school	3.33	2.68	4.14
Secondary	2.53	2.13	2.99
Tertiary	Ref		
**Marital Status**			
Married	Ref		
Single	1.01	0.84	1.23
Divorce/widow/er	1.09	0.76	1.58
**Occupation**			
Government	1.38	1.01	1.89
Private	1.80	1.30	2.37
Self employed	1.86	1.42	2.45
Unpaid worker/homemaker	0.73	0.47	1.13
Retiree	Ref		
**Residential**			
Urban	Ref		
Rural	1.12	0.96	1.28

## DISCUSSION

The present study revealed that in 2015 more than one-fifth of Malaysian adults were current smokers, which is comparable to the global smoking prevalence in 2014 of 22.5%^16^. Similar rates were reported in Vietnam (23.8%)^17^ and Thailand (23.7%)^18^, but higher rates were reported in India (34.5%)^19^, Ukraine (28.8%)^20^ and China (28.1%)^21^. Nevertheless, the smoking prevalence in Malaysia was still high when compared to developed countries such as Singapore (16%)^22^ and Australia (12.8%)^23^. The difference in the prevalence across countries might be due to disparities in the socio-economics^24^, culture^25^, tobacco legislation^26^ and taxation between countries^24^. The ratio of Malaysian male to female smokers (31:1)^12-13^ was similar to that of Vietnam (34:1)^17^, but lower than that of Thailand (22:1)^18^ and China (5:1)^21^. Such a high male to female ratio could be due to prevailing social norms that are not conducive to females smoking^27^.

Approximately half of male adults of Malay, ‘Other indigenous’ and ‘Others’ classifications were current smokers and this proportion agrees with figures reported from the previous NHMS surveys^10-12^ and GATS 2011^13^. Multivariable analysis also revealed that these ethnic groups were more likely to smoke compared to the Chinese. However, the association between ethnicity and smoking may also be attributed to many other potential intrinsic and extrinsic factors, such as perceived societal norms, culture, and religion^28-31^ that need to be further explored in future studies.

The lower likelihood of smoking among Malaysian elderly could be attributed to several reasons. First, it was most likely that the risk of premature death was higher among smokers^32^, while non-smokers tend to survive to old age. The elderly tend to have more health problems due to advancing age and this may have a big influence in encouraging their desire to cease smoking, compared to their younger, healthier counterparts^33^. Moreover, their health condition may require them to visit health facilities for treatment more frequently^34,35^ and so indirectly increase their exposure to anti-smoking messages^30^. Since older people tend to be more receptive to health messages^36^, this might also lead them to quit smoking^37^. In contrast, an increase by 2.3% in the proportion of current smokers among adolescents aged 15-24 years was observed from 201113 to 2015, and this phenomenon is indeed alarming as it indicates that the younger and more susceptible generations are initiating smoking. They are most likely influenced by smoking adults or peers around them^38-39^ or by the relentless promotion activities by tobacco companies, which are now mainly aimed at the adolescent market^40,41^. Properly designed studies on smoking among Malaysian adolescents are much needed to identify the determinants of smoking so as to develop and implement effective anti-smoking policies.

The present study findings are consistent with those of studies conducted elsewhere^39,42-44^, where individuals with a higher level of educational attainment were less likely to smoke. This might be due to respondents with higher educational attainment having better knowledge about and more receptive to information on health^45,46^. They also have better coping skills in stress management^47^, and so less likely to resort to tobacco products to alleviate stress.

On the other hand, the likelihood to smoke was lower among those at the lowest or highest income level quintiles compared to those at the middle income quintiles. These findings were contrary to those reported in a previous study 2006^39^, where the investigators found that the likelihood of becoming a smoker was higher among those in the low-income group. The drastic increase in the price of tobacco products, as a result of the increase in excise tax from 0.12 cents/stick in 2006 to 0.25 cents/stick in 2014^48^, may have reduced their affordability among smokers of lower income, since it has been shown that a 10% price increment on tobacco products can cause a 4% reduction in sales^49^. The proportion of current smokers decreased significantly from 32.0% (95% CI: 27.3-37.1) in the GATs study to 19.3% (95% CI: 17.22-1.1) in 2015. However, the present findings also indicate that the price increment in tobacco products has only affected those with low income but not those in the middle income quintile groups (Quintiles 2-4). In contrast, adults in the highest income quintile were less likely to smoke and this can be substantiated by findings of other studies^49,50^, where a higher income level was a protective factor against smoking, since these people were more likely to be literate^51^, and therefore may have better knowledge and awareness about health^52^.

The present study did not find a significant association between marital status, locality and smoking, among Malaysian adults. These findings were not in line with previous studies, such as that of Lim et al. (2013)^39^ who reported that divorced males or widowers were more likely to smoke and that of Lindström and Isacsson (2010)^53^ who also found a higher likelihood of smoking among unmarried, divorced or stayed-alone adults. Therefore, the ‘marriage protection’ theory that posits that married individuals receive more social and psychological support to enable them to quit smoking, and the ‘marriage selection’ theory that states that healthier non-smokers are more likely to get and stay married, were not applicable to the Malaysian population^54^.

In terms of locality, the present findings showed no significant association between locality and smoking, and this contradicted the findings reported by Lim et al. 2013^39^, and several studies from other countries^55-57^, that revealed that rural residents were more likely to smoke. Despite the fact that many studies have demonstrated that urban residents were more likely to be educated and more often exposed to anti-smoking campaigns, and therefore less likely to smoke compared to their rural counterparts^58,59^, the increased level of stress due to work and cost of living as well as other intra- and inter-personal factors may have resulted in a similar likelihood of smoking as the rural residents^60,61^. These findings warrant detailed investigation from an intra-personal (knowledge and attitude) and inter-personal (urbanisation effect) aspects to identify the actual contributing factors.

The present study also found that retirees were less likely to smoke compared to government employees, private sector employees and the self-employed. The possible explanations for such findings are that respondents who work encounter more physical (e.g. noise, second-hand smoke, long working hours, and shift work), psychosocial and mental stress arising from a lack of job autonomy (e.g. lack of control of job autonomy or of promotional prospects)^62-66^, and therefore the likelihood of stress-induced smoking may be higher.

The high prevalence of cigarette use (90%), found in the present study, is comparable to that of the Philippines (97.8%)^67^ and China (94.8%)^68^, but in Thailand^18^ and India^25^ hand-rolled tobacco is the most commonly consumed tobacco product. The higher preference for cigarettes over other tobacco products may be due to their quality as they are designed to mask harshness, provide a particular taste sensation, and increase nicotine delivery^69-70^. In addition, marketing strategies by tobacco companies to increase and sustain cigarette consumption might also contribute to its popularity. Besides cigarettes, the use of smokeless tobacco has increased almost 16-fold from 0.7% in 2011^13^ to 10.9% in 2015. This tremendous increase may be attributed to e-cigarette use, which is classified as smokeless tobacco in the present study, as e-cigarettes have been well accepted by Malaysian smokers^70^. In view of the rapid rise in smokeless tobacco use, particularly e-cigarette use, it is timely to review the Control of Tobacco Product Regulation to include smokeless tobacco products. Almost half of the Malaysian population used 10 or less sticks of cigarettes per day, and this rate of consumption is similar to 10 years ago^39^. This indicates that the nicotine addiction level among Malaysian smokers can be considered low, and therefore the chances of success for smoking cessation programmes are higher. On the other hand, the present study also revealed the proportion of Malaysian adults who smoke more than 21 sticks per day had increased by 18% from 2006^12^. Further studies are necessary to identify the actual contributing factors.

Our study indicates that the smoking prevalence among Malaysian male adults is still high and that there was a substantial increase in the proportion of smokers among young adults. Moreover, the prevalence of smokeless tobacco use and number of cigarettes smoked per day have increased. These findings support the need for a reassessment of the current anti-smoking policies in terms of their planning, implementation and evaluation.

The comparison of smoking prevalence with the previous nationwide surveys of 1996, 2006 and 2011, demonstrate little progress on smoking control in Malaysia over the last three decades. Only a significant reduction in smoking prevalence was observed among the respondents from the older age group (65 years and over), unpaid workers, homemakers and retirees. Some reductions were also observed among males, respondents from Chinese descendants, and those who are widow/widower/divorcee. The comparisons of smoking prevalence among Malaysian adults aged 15 years and over for 2011 and 2015, show a significant reduction in smoking among the lowest income group. The study also shows increasing proportion of smoking among the younger age group (15-24 years), while for females, those with no formal education, and those residing in rural areas, smoking prevalence has not significantly changed during the last three decades. However, the comparison should be interpreted cautiously in view of differences in the definition of ‘current smoker’ for each survey (Table 5).

**Table 5:** Current smoking status* among Malaysian adults 1996-2015

*Social-demographic characteristics*	*Smoking prevalence*			
	*1996*	*2006*	*2011*	*2015*
**OVERALL**	24.8 (24.1-25.4)	21.5(21.0-22.0)	23.1(21.2-25.2)	22.8(21.9-23.8)
**Location**				
Urban	21.3(20.5-22.2)	19.0(18.3-19.6)	22.4(19.9-24.1)	21.2(20.1-22.4)
Rural	28.0(27.1-29.0)	26.2(25.4-27.0)	24.2(21.9-26.6)	27.9(26.3-29.6)
**Sex**				
Male	48.2(47.1-49.4)	46.4(45.5-47.4)	43.9(40.6-47.3)	43.0(41.4-44.6)
Female	3.4(3.1-3.8)	1.6(1.5-1.8)	1.0(0.7-1.6)	1.4(1.1-1.8)
Age group**				
15-24	20.9(19.5-22.3)	23.3(22.0-24.3)	16.6(13.5-20.3)	19.5(17.8-19.5)
25-44	24.2(23.3-25.0)	23.4(22.7-24.1)	28.9(25.9-32.0)	28.3(26.9-29.8)
45-64	26.8(25.7-27.9)	19.4(18.6-20.1)	22.5(19.7-25.7)	20.1(19.0-21.3)
65+	25.8(23.9-27.8)	16.6(15.3-17.9)	13.9(10.3-18.5)	11.5(9.8-13.4)
**Ethnicity**				
Malays	27.5(26.6-28.4)	24.0(23.4-24.7)	24.3(21.9-27.0)	24.7(23.6-25.9)
Chinese	18.8(17.7-19.9)	16.2(15.3-17.2)	15.3(11.9-19.4)	14.2(12.7-15.9)
Indians	15.9(14.2-17.9)	13.7(12.4-15.0)	19.2(13.9-26.0)	16.5(14.0-19.4)
Other Bumiputras	29.7(28.2-31.3)	24.8(23.4-26.2)	29.9(25.1-35.2)	25.8(23.4-28.4)
**Marital status**				
Single	26.1(24.7-27.6)	26.7(25.7-27.8)	24.9(21.7-28.5)	23.6(21.9-25.3)
Married	24.8(24.1-25.5)	21.2(20.7-21.7)	22.9920.6-25.3)	23.8(22.8-24.9)
Widow/widower/divorcee	16.2(14.7-17.9}	9.3(8.2-10.3)	12.1(8.8-16.4)	9.7(8.0-11.8)
**Education level**				
No formal education	21.(20.1-22.9)	15.3(14.1-16.6)	19.4(15.6-23.9)	21.5(17.7-26.0)
Primary	27.7(26.7-28.8)	21.9(21.1-22.7)	23.8(20.6-27.4)	25.2(23.3-27.2)
Secondary	25.(24.4-26.2)	24.1(23.4-24.8)	25.1(22.3-28.1)	25.8(24.5-27.1)
Tertiary	17.1(15.4-18.9)	14.2(13.0-15.4)	15.3(11.6-19.9)	14.9(13.6-16.3)
**Occupation**				
Government/semi-government	28.7(26.8-30.6)	23.1(21.7-24.6)	25.5(20.8-30.9)	23.1(20.6-25.8)
Private	31.3(30.0-32.6)	29.9(29.1-30.8)	34.3((30.6-38.2)	31.7(29.9-33.6)
Self-employed	39.2(37.7-40.7)	35.6(34.4-36.7)	44.6(39.3-50.0)	35.4(33.2-37.6)
Unpaid worker/homemaker	8.4(7.8-9.0)	6.7(6.3-7.0)	5.4(4.1-7.6)	2.1(1.5-2.90)
Retiree	32.9(29.0-37.1)		17.7(11.7-25.8)	19.3(16.0-23.2)
**Income level (a)**				
less than RM 1000	25.7(24.8-26.5)	24.2(23.4-25.0)		
RM 1000-RM1999	25.2(24.1-26.3)	23.1(22.3-24.0)		
RM 2000-RM2999	21.6(20.0-23.2)	20.5(19.4-21.6)		
RM 3000-RM3999	21.2(19.1-23.4)	19.3(17.8-20.9)		
RM 4000-RM4999	22.7(19.8-25.9)	16.6(14.7-18.6)		
RM 5000+	21.1(18.3-24.1)	14.9(13.6-16.2)		
**Income level (b)**				
Quintile 1			28.7(23.8-34.1)	16.5(14.6-18.5)
Quintile 2			27.8(22.8-33.4)	26.8(24.8-28.9)
Quintile 3			27.4(23.6-31.5)	25.1(23.4-26.9)
Quintile 4			19.7(15.9-24.2)	25.5(23.7-27.4)
Quintile 5			16.7(13.8-20.0)	19.3(17.7-21.1)

* **Definition of current smoker** (a) Respondent who reported to be smoking at the time of the survey (b) Respondent who reported to have smoked 100 or more cigarettes in lifetime and smoked daily or some days in the past one month (c) Smoker who daily or occasionally smokes any tobacco product

** **Age Group** For 1996 and 2006, only adults age 18 years and over were included in the study

***Income level Quintile 1 is the lowest and Quintile 5 is the highest

The present study is subject to some limitations which are noted here. First, the study being cross-sectional, allows the establishment of association only between socio-demographic variables and smoking. Secondly, smoking status was determined via self-report without objective biochemical verification. This could have contributed to under or over reporting of smoking^71^. Thirdly, intra-personal factors, such as knowledge, attitude^46^, stress level^47^ and depression^48^, which have been identified as determinants of smoking were not investigated in the present study. However, the representativeness and adequacy of sample size, as well as the high response rate allow generalization of the findings to the Malaysian population.

## CONCLUSIONS

The present findings revealed that the smoking prevalence among Malaysian male adults was still high and that there was a dramatic increase among the young adults aged 15-24 years. Also the rising prevalence of smokeless tobacco use and number of cigarettes smoked are alarming. These findings warrant immediate actions for planning, implementation and improvement of anti-smoking policies, specially targeted at these sub-populations to reduce the morbidity, mortality and economic burden of the country.

## References

[1] Beaglehole R, Bonita R, Horton R, Adams C, Alleyne G, Asaria P (2011). Priority actions for the non communicable disease crisis. Lancet.

[2] Peto R, Boreham J, Lopez AD, Thun M, Health C (1992). Mortality from tobacco in developed countries: indirect estimation from national vital statistics. Lancet.

[3] Peto R (1994). Smoking and death: the past 40 years and the next 40. Br Med J.

[4] Disease Control Division, Ministry of Health (2003). Clinical Practice Guidelines. Treatment of Tobacco smoking and dependence 2002.

[5] Institute of Public Health, Ministry of Health Malaysia 2004 Malaysian Burden of Disease and Injury Study, Health Prioritization: Burden of Disease Approach.

[6] Institute of Public Health, Ministry of Health Malaysia 2011 Malaysian Burden of Disease and Injury Study, Health Prioritization: Burden of Disease Approach.

[7] Lim KH, M Fadhli Y, Omar M, Rosnah R, M, Nazaruddin B, Sumarni MG Technical Report. Evaluation of effectiveness of implementation of “Komuniti Sihat Perkasa Negara” (KOSPEN) Programme in Malaysia - Phase 1.

[8] Malaysia Act (2008). Food Act (The Control of Tobacco Product Regulations 2004).

[9] World Health Organization WHO Framework Convention on Tobacco Control.

[10] Institute of Public Health, Ministry of Health Malaysia National Health and Morbidity Survey, 1986 -1987, Volume IV - Cigarette Smoking.

[11] Institute of Public Health, Ministry of Health Malaysia 1996 National Health and Morbidity Survey. 1997 Volume 15, Smoking.

[12] Institute of Public Health, Ministry of Health Malaysia 2008 The Third National Health and Morbidity Survey - Smoking, 2006.

[13] Institute for Public Health (2012). Report of the Global Adult Tobacco Survey (GATS). Malaysia 2011.

[14] Department of Statistics, Malaysia (2011). Buletin Perangkaan KKLW.

[15] Archer KJ, Lemeshow S, Hosmer DW (2007). Goodness-of-fit tests for logistic regression models when data are collected using a complex sampling design. Comput Stat Data An.

[16] Gowing LR, Ali RL, Allsop S, Marsden J, Turf EE, West R (2015). Global statistics on addictive behaviours: 2014 status report. Addic.

[17] Ministry of Health Viet Nam (2010). Global Adult Tobacco Survey (GATS) Viet Nam.

[18] Ministry of Public Health Global adult tobacco survey (GATS): Thailand country report.

[19] Ministry of Health and Family Welfare Global Adult Tobacco Survey (GATS) Indian Fact Sheet (2009-2010).

[20] Palipudi KM, Gupta PC, Sinha DN, Andes LJ, Asma S (2012). Social Determinants of Health and Tobacco Use in Thirteen Low and Middle Income Countries: evidence from Global Adult Tobacco Survey. PLoS ONE.

[21] Qiang L, Hsia J, Yan Y, Lin X, Xiao WC, Feng G, Yang G Global Adult Tobacco Survey (GATS) China 2010 Country Report.

[22] Picco L, Subramaniam M, Abdin E, Vaingankar JA, Chong SA (2012). Smoking and nicotine dependence in Singapore: findings from a cross-sectional epidemiological study. Ann Acad Med Singapore.

[23] Australia Institute of Health and Welfare National Drug Strategy Household Survey 2013. Tobacco smoking in the general population.

[24] Siahpush M, Mcneill A, Hammond D, Fong GT (2006). Socioeconomic and country variations in knowledge of health risks of tobacco smoking and toxic constituents of smoke: results from the 2002 International Tobacco Control (ITC) Four Country Survey. Tob Cont.

[25] Satterlund TD, Antin TMJ, Lee JP, Moore RS (2009). Cultural factors related to smoking in San Francisco’s Irish bars. J Drug Educ.

[26] John Lee T, Glantz SA, Millett C (2011). Effect of Smoke-Free Legislation on Adult Smoking Behaviour in England in the 18 Months following Implementation. PloS one.

[27] Graham H (1996). Smoking prevalence among women in the European Community 1950-1990. Soc. Sci. Med.

[28] Ugen S (2003). Bhutan: the world’s most advanced tobacco control nation?. Tob Cont.

[29] França LR, Dautzenberg B, Falissard B, Reynaud M (2009). Are social norms associated with smoking in French university students? A survey report on smoking correlates. Subst Abuse Treat Prev.

[30] Mohammadnezhad M, Tsourtos G, Wilson C, Ratcliffe J, Ward P (2015). Understanding Socio-cultural Influences on Smoking among Older Greek-Australian Smokers Aged 50 and over: Facilitators or Barriers? A Qualitative Study. Int J Environ Res Public Health.

[31] Yong HH, Hamann SL, Borland R, Fong GT, Omar M (2009). Adult smokers’ perception of the role of religion and religious leadership on smoking and association with quitting: A comparison between Thai Buddhists and Malaysian Muslims. Soc Sci Med.

[32] World Health Organization Media Centre Tobacco Fact sheet.

[33] Grotvedt L, Stavem K (2005). Association between age, gender and reasons for smoking cessation. Scan J Public Health.

[34] Krishnaswamy S, Subramaniam K, Low WY, Aziz JA, Indran T, Ramachandran P (2009). Factors contributing to utilization of health care services in Malaysia: A population-based study. Asia Pac J Public Health.

[35] Institute of Public Health (2015). National Health & Morbidity Survey 2015. Health Care Demand.

[36] Kendig H, Helme R, Teshuva K (1996). Health Status of Older People Project: preliminary findings from a survey of the health and lifestyles of older Australians.

[37] Abdullah AS, Driezen P, Anne Quah CK, Nargis N, Fong GT (2015). Predictors of smoking cessation behavior among Bangladeshi adults: findings from ITC Bangladesh survey. Tob Induc Dis.

[38] Polanska K, Jurewicz J, Hanke W (2015). Smoking and alcohol drinking during pregnancy as the risk factors for poor child neurodevelopment – A review of epidemiological studies. Int J Occup Med Environ Health.

[39] Lim KH, Ghazali S, Kee C, Lim KK, Chan Y, Teh H (2013). Epidemiology of Smoking Among Malaysian Adult Males: Prevalence and Associated Factors. BMC Public Health.

[40] Lim Khor Yoke, Kin Foong Tobacco Advertising And Smoking Amongst Adolescents: A Qualitative Study.

[41] Institute for Public Health (IPH) (2016). Tobacco & E-Cigarette Survey Among Malaysian Adolescents (TECMA).

[42] Kaleta D, Makowiec-Dabrowska T, Dziankowska-Zaborszczyk E, Fronczak A (2012). Prevalence and socio-demographic correlates of daily cigarette smoking in Poland: Results from the Global Adult Tobacco Survey (2009–2010). Int J Occup Med Environ Health.

[43] Lim KH, Jasvindar K, Cheong SM, Ho BK, Lim HL, Teh CH (2016). Prevalence of smoking and its associated factors with smoking among elderly smokers in Malaysia: findings from a nationwide population-based study. Tob Induc Dis.

[44] Jha P, Ramasundarahettige C, Landsman V, Rostron B, Thun M, Anderson RN (2012). 21st-century hazards of smoking and benefits of cessation in the United States. N Engl J Med.

[45] Gilman SE, Martin LT, Abrams DB, Kawachi I, Kubzansky L, Loucks EB (2008). Educational attainment and cigarette smoking: a causal association?. Int J Epidemiol.

[46] Cowell AJ (2006). The relationship between education and health behavior: some empirical evidence. Health Econ.

[47] Slopen N, Kontos EZ, Ryff CD, Ayanian JZ, Albert MA, Williams DR (2013). Psychosocial stress and cigarette smoking persistence, cessation, and relapse over 9–10 years: a prospective study of middle-aged adults in the United States. Cancer Causes Control.

[48] Ministry of Plantation and Commodities Industries (MPIC) TOBACCO: Tobacco industry taxed to the precipice.

[49] Reddy KS, Gupta PC (2004). Policy intervention: Taxation in report on tobacco control in India.

[50] Gilmore ABC, McKee M, Telishevska M, Rose R (2001). Epidemiology of smoking in Ukraine. Prev Med.

[51] Huisman M, Kunst AE, Mackenbach JP (2005). Inequalities in the prevalence of smoking in the European Union: comparing education and income. Prev Med.

[52] Liu YB, Liu L, Li YF, Chen YL (2015). Relationship between Health Literacy, Health-Related Behaviors and Health Status: A Survey of Elderly Chinese. Int. J. Environ. Res. Public Health.

[53] Lindström M, Isacsson SO, Malmö Shoulder-Neck Study Group (2002). Smoking cessation among daily smokers, aged 45-69 years: a longitudinal study in Malmö, Sweden. Addiction.

[54] Schoenbom C (2004). Marital status and health: United States, 1999–2002, advance data from vital and health statistics.

[55] Aekplakorn W, Hogan MC, Tiptaradol S, Wibulpolprasert S, Punyaratabandhu P, Lim SS (2008). Tobacco and hazardous or harmful alcohol use in Thailand: Joint prevalence and associations with socioeconomic factors. Addict Behav.

[56] Lim SG, Chung WJ, Kim HJ, Lee SM (2010). The influence of housing tenure and marital status on smoking in South Korea. Health Policy.

[57] Li Q, Hsia J, Yang GH (2011). Prevalence of smoking in China in 2010. N Engl J Med.

[58] Doescher MP, Jackson JE, Jerant A, Hart LG (2006). Prevalence and Trends in Smoking: A National Rural Study. J. Rural Health.

[59] National Rural Health Alliance Inc (2013). Smoking and Rural Health, Fact Sheet.

[60] Patel V, Leon D, Walt G (2001). Poverty, inequality, and mental health in developing countries. Poverty, inequality and health: an international perspective.

[61] Srivastava K (2009). Urbanization and mental health. Ind Psychiatry J.

[62] Gamperiene M, Nygard J, Sandanger I, Warsted M, Bruusgaard D (2006). The impact of psychosocial and organizational working conditions on the mental health of female cleaning personnel in Norway. J Occup Med Toxicol.

[63] Elovainio M, Kivimäki M, Steen N, Vahtera J (2004). Job decision latitude, organizational justice and health: multilevel covariance structure analysis. Soc Sci Med.

[64] Androniki P, Elizabeth M, Derek W (2001). Control and role conflict in food service providers. International Journal of Hospitality Management.

[65] Nebot M, Lόpez MJ, Ariza C, Pérez-Rίos M, Fu M, Schiaffino A (2008). Impact of the Spanish smoking law on exposure to secondhand smoke in offices and hospitality venues: Before-and-After Study. Environ Health Perspect.

[66] Cenko C, Pisaniello D, Esterman A (2004). A study of environmental tobacco smoke in South Australian pubs, clubs and cafes. Int J Environ Health Res.

[67] Global Adult Tobacco Survey (GATS) (2009). Philippines country report.

[68] China Control Disease Centre Global Adult Tobacco Survey (GATS). China 2010 Country Report.

[69] Rees VW, Kreslake JM, Geoffrey Wayne GF, O Connor RJ, Cummings KM, Gregory N, Connolly GN (2012). Role of Cigarette Sensory Cues in modifying puffing topography. Drug Alcohol Depend.

[70] Hammond D, Fong GT, Cummings KM, Hyland A (2005). Smoking Topography, Brand Switching, and Nicotine Delivery: Results from an in vivo study. Cancer Epidemiol Biomarkers Prev.

[71] Gravely S, Fong GT, Cummings KM, Yan M, Anne Quah CK, Borland R (2014). Awareness, Trial, and Current Use of Electronic Cigarettes in 10 Countries: Findings from the ITC Project. Int. J. Environ. Res. Public Health.

[72] Klein J, Thomas RK, Sutter EJ (2007). Self-reported smoking in online surveys: prevalence estimate validity and item format effects. Med Care.

[73] Lim KH, Sumarni MG, Amal NM, Hanjeet K, Wan Rozita WM, Norhamimah A (2009). Tobacco use, knowledge and attitude among Malaysians age 18 and above. Trop Biomed.

[74] Taylor G, Mcneil A, Girling A, Farley A, Lindson-Hawley N, Aveyard P (2014). Change in mental health after smoking cessation: systematic review and meta analysis. BMJ.

